# Case Report: Minimally invasive removal of impacted supernumerary teeth using digital 3D-printed surgical guides: a case series

**DOI:** 10.3389/fsurg.2025.1653013

**Published:** 2025-11-17

**Authors:** Qilin Chen, Zhiwei Li, Zhenzhen Li, Wenpin Xu, Zijing Yu, Jian Wang

**Affiliations:** 1 Department of Stomatology, Taihe Hospital, Hubei University of Medicine, Shiyan, Hubei, China; 2 Department of Cardiac Function, Cardiovascular Diagnosis and Treatment Center, Taihe Hospital, Hubei University of Medicine, Shiyan, Hubei, China

**Keywords:** supernumerary teeth, digital 3D printing, surgical guide, minimally invasive surgery, oral surgery

## Abstract

The surgical management of impacted supernumerary teeth (ST) remains challenging because of their hidden position and close proximity to vital anatomical structures. These guides offered accurate intraoperative guidance and minimized both bone removal and soft-tissue dissection. A standardized digital workflow that integrates cone-beam computed tomography (CBCT) and intraoral scans was employed to create patient-specific guides that precisely dictated the osteotomy location. The guides provided critical intraoperative guidance and minimized the extent of bone removal and soft tissue dissection. This accurate and focused approach contributed to observed reductions in postoperative discomfort and swelling, supporting an overall favorable recovery profile in all cases. The application of 3D-printed surgical guides enables a highly precise and minimally invasive approach for the removal of impacted ST, thereby improving postoperative recovery. Furthermore, this technique shows considerable potential for broader application in oral and maxillofacial surgery by ensuring precise localization, reducing complications, and enhancing operative efficiency.

## Introduction

Supernumerary teeth (ST) refer to additional teeth that develop beyond the normal dental complement. These rare dental abnormalities result from disruptions in odontogenesis and are often linked to environmental and genetic factors (1–3). The most commonly reported location of ST is the anterior maxilla, especially the midline (mesiodens), followed by the premolar and molar areas. However, the prevalence of ST varies among populations (4). ST exhibit various morphological types, including supplementary, tuberculate, and conical forms. Clinically, the presence of ST can lead to significant issues, such as crowding, delayed eruption, or displacement of adjacent teeth, which negatively affect dental function and appearance (5). Efficient localization is essential because the frequently concealed and embedded position of ST complicates traditional methods, making them more invasive. A recent systematic review highlights that 3D printing technologies enable less invasive procedures for benign jaw lesions by facilitating precise osteotomies and reducing operating time and complications (6). Digital 3D-printing guidance minimizes bone removal and tissue trauma, thereby shortening surgical duration.

Digital 3D-printed surgical guides are personalized intraoral devices rooted in the core principle of anatomical matching and preoperative planning integration—they translate 3D imaging data into physical navigation tools, bridging the gap between virtual planning and intraoperative execution. Fabrication follows a standardized digital workflow: (1) acquisition of high-resolution 3D data—CBCT for bony structures and intraoral scans for dental and gingival contours—exported in DICOM and STL formats; (2) data registration, 3D reconstruction, and guide design using specialized software (e.g., Mimics, 3-Matic); and (3) fabrication with biocompatible materials (photopolymer resin or titanium) through additive manufacturing, followed by post-processing (cleaning, curing, sterilization). This versatile guide plate effectively addresses the core challenges faced by traditional manual techniques through its ability to enhance surgical precision, minimise trauma, and standardise procedures.

This case series introduces a minimally invasive approach for the extraction of multiple impacted maxillary and mandibular supernumerary teeth using digitally designed and 3D-printed surgical guides.

## Shared workflow

A complete CBCT examination was performed using a 3D CBCT scanner (field of view: 10 × 10 cm, voxel size: 0.125 mm. Orthophos SL 3D, Sirona Dental Systems GmbH, Germany), and the data were exported in DICOM format. An intraoral scan was conducted using an intraoral scanner (i500, Medit Corp, Seoul, Korea), and the data were exported in STL format.

The DICOM and STL files were imported into Mimics 25.0 and 3Matic 16.0 software for 3D reconstruction. CBCT data was used to reconstruct bony structures and supernumerary teeth, while intraoral scan data was used to reconstruct the dental crowns and gingival contour, achieving registration with a deviation of <0.1 mm. Surgical guides were designed via CAD technology with the following parameters: material designated as biocompatible photopolymer resin (Real Maker M, Zhuzhen Electronic Technology, Yiwu, China), thickness of 2.0 mm, and a semi-circular operative window (diameter: 5–8 mm, tailored to supernumerary tooth size) aligned with the target tooth. The guide's occlusal/palatal surface was designed to fit the anatomical contour of adjacent teeth for retention.

Guides were fabricated using a Formlabs Form 3B + resin 3D printer (layer height: 50 μm, printing speed: 10 gypsum models/30 min. Real Maker M, Zhuzhen Electronic Technology, Yiwu, China) with biocompatible dental photopolymer resin (Real Maker M, Zhuzhen Electronic Technology, Yiwu, China). Post-printing, guides underwent isopropyl alcohol cleaning (10 min), UV curing (Form Cure, 20 min at 60°C), and sterilization via autoclaving (134°C, 20 min). No manufacturing complications—such as fractures, misalignments, or misprints—were observed in any guide, as verified by preoperative fit testing on 3D-printed dental models.

Preoperative examinations were conducted to exclude any surgical contraindications. Each patient was scheduled for supernumerary tooth extraction in an outpatient setting under local anesthesia. Written informed consent for the surgical procedure was obtained from all patients.

## Case presentation

### Case 1

A 20-year-old female patient was incidentally diagnosed with supernumerary teeth in the right mandible during a routine periodontal examination 1 week earlier. She had not received any prior treatment and reported no discomfort.

**Figure 1:**
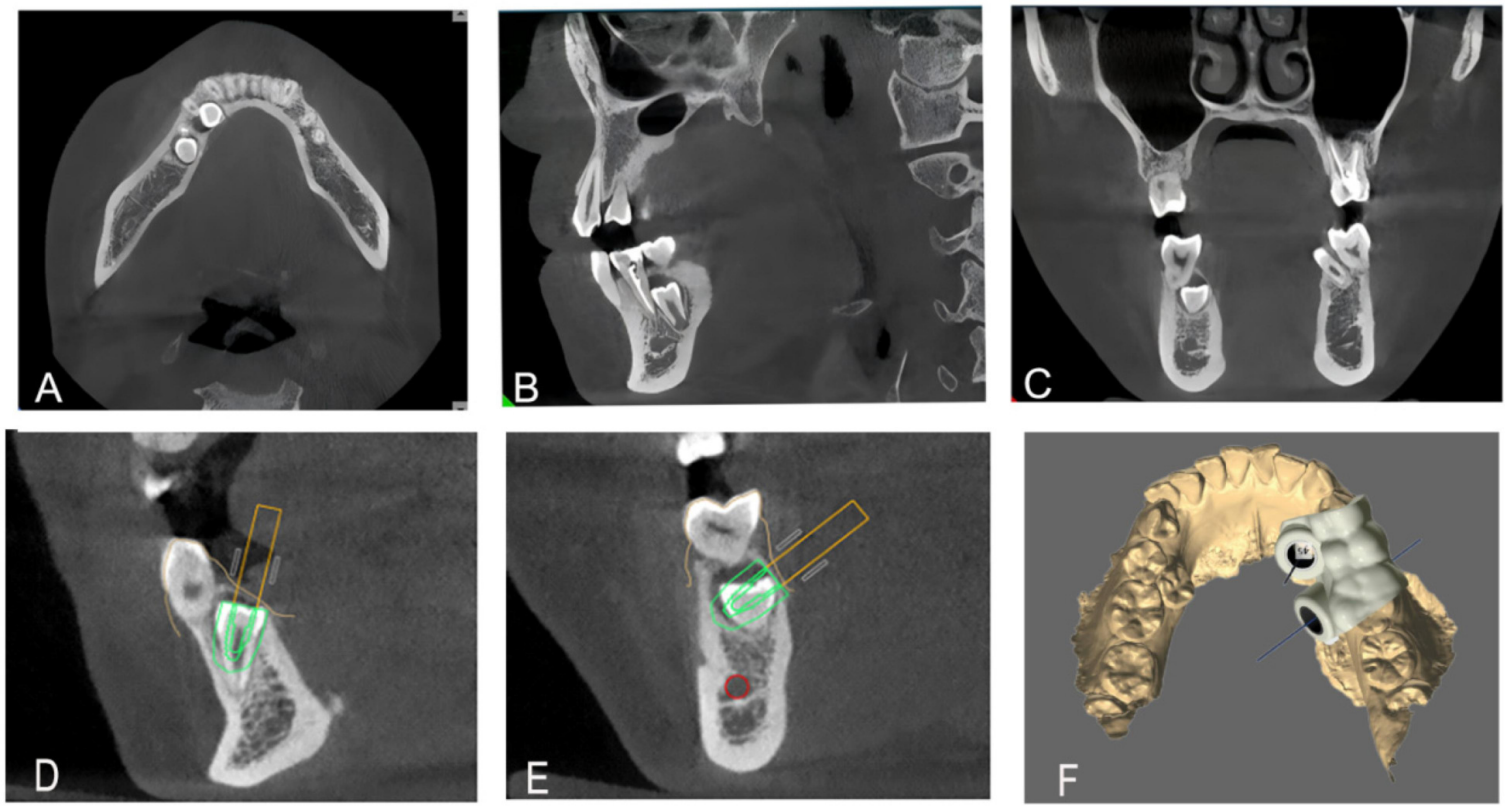
Preoperative CBCT examination revealed the position of the supernumerary teeth in the axial **(A)**, sagittal **(B)**, and coronal planes **(C)**, **(D–F)**. Digital 3D-printed guide design, guide design.

Clinical examination revealed no abnormalities in the posterior right mandibular region. The patient presented with a fully developed permanent dentition and healthy periodontium. However, according to the CBCT scan, an extra tooth was found respectively in the root area of teeth 43/44 and 45/46 (Figure 1).

#### Diagnostic reasoning

Initial routine periodontal radiography suggested an opaque mass in the root area of 43/44, but the 2D image failed to clarify its relationship with the inferior alveolar nerve. Subsequent CBCT confirmed it as a supernumerary tooth by showing a complete crown-root structure, distinct from the adjacent teeth, and no connection to the dental lamina of permanent teeth.

#### Diagnosis

Diagnosis: Two impacted mandibular supernumerary teeth.

#### Treatment progress

**Figure 2:**
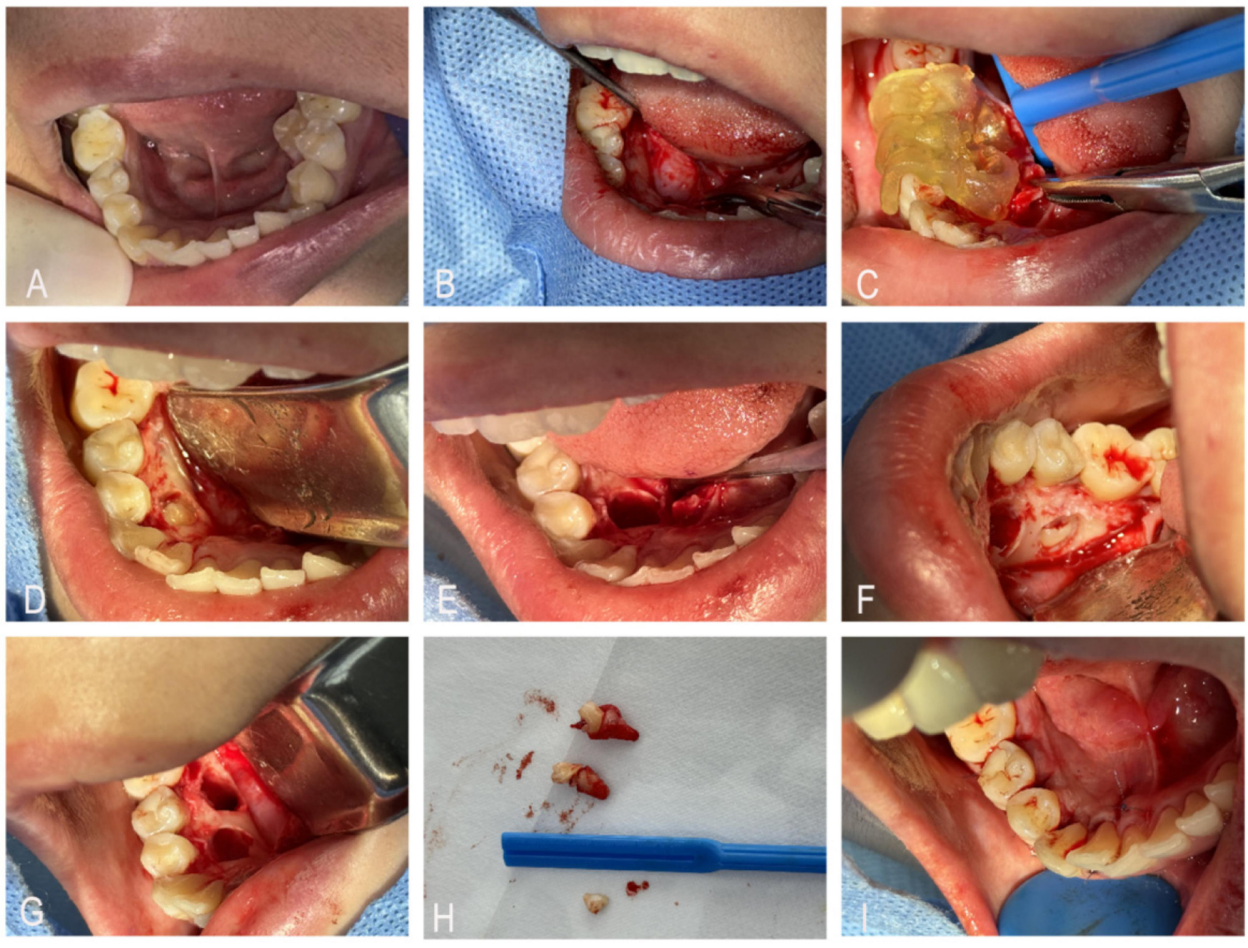
**(A)** Preoperative intraoral view showing no visible supernumerary teeth. **(B)** Incision and flap elevation to expose the bone plate. **(C)** Placement of the surgical guide. **(D–H)** Bone removal using a high-speed turbine through the guide's positioning holes, followed by stepwise deepening and enlargement to expose the tooth crown, sectioning part of the crown to eliminate resistance, and extracting the supernumerary tooth. **(I)** Suturing and closure of the incision.

The surgical field was disinfected with povidone-iodine and covered with sterile drapes. A lingual incision was made between teeth 43 and 46, and a mucoperiosteal flap was elevated to expose the underlying bone. The sterilized digital 3D-printed surgical guide was then fixed in place. The guide achieved retention via passive fit to the occlusal and lingual surfaces of teeth 43–46—its contoured internal surface matched the anatomical morphology of the dental crowns, ensuring stable positioning without additional fixation devices (e.g., screws). A pre-surgical fit check confirmed no lateral or vertical displacement under gentle pressure. Using a high-speed handpiece with a 45-degree angled bur (grain size: 80 μm, diameter: 1.2 mm), bone removal was performed according to the predesignated points on the surgical guide. After bone removal, the crown of the impacted supernumerary tooth was exposed. The crown was sectioned to remove the obstruction, and the supernumerary tooth was extracted. The surgical area was thoroughly irrigated with a 0.9% saline solution. The incision was closed with 4-0 absorbable polyglycolic acid (PGA) sutures, and pressure was applied for 40 min to control bleeding. After hemostasis, the patient received postoperative instructions and was discharged (Figure 2). The patient reported mild soreness within 24 h, relieved by a single dose of ibuprofen (200 mg). No pain, numbness, or paresthesia occurred during follow-up. The timeline presentation of Case 1 has been presented in Supplementary Table S1.

### Case 2

A 13-year-old female patient was incidentally found to have a supernumerary tooth in the left mandible during an external radiographic examination conducted 15 days earlier. Oral examination showed a permanent dentition with mild crowding. CBCT revealed a supernumerary tooth located apical to the roots of teeth 34 and 35, positioned horizontally between them. CBCT revealed a supernumerary tooth located apical to the roots of teeth 34 and 35, positioned horizontally between their roots.

#### Diagnostic reasoning

External radiography demonstrated a radiopaque lesion between the roots of 34 and 35. CBCT showed its horizontal orientation and absence of root resorption in adjacent teeth, thereby excluding odontoma.

#### Diagnosis

Diagnosis: An inter-radicular supernumerary tooth between teeth 34 and 35.

#### Treatment progress

**Figure 3:**
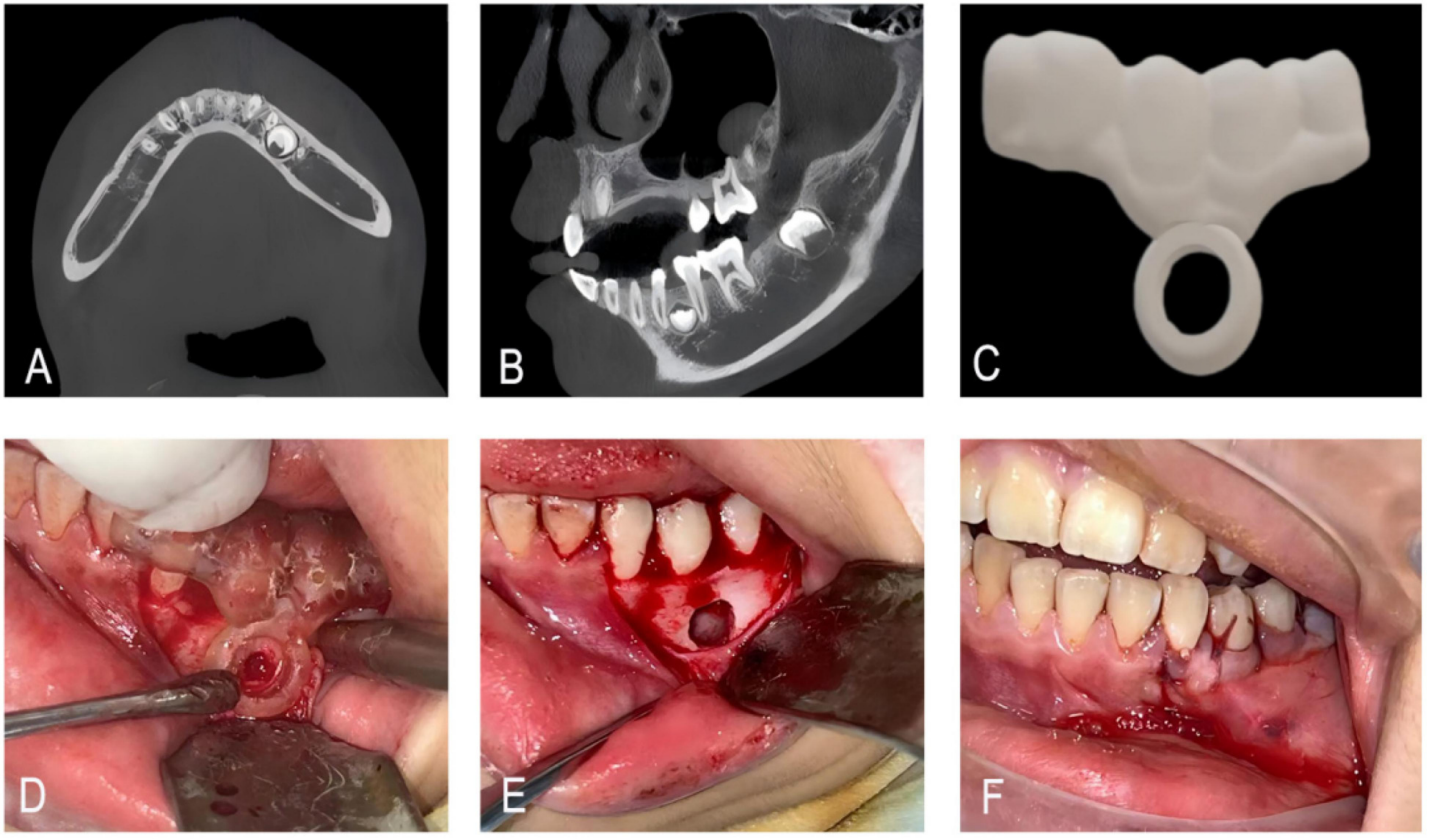
**(A,B)** CBCT showing a supernumerary tooth apical to the roots of teeth #34 and #35. **(C)** Guide plate designed by 3D printing. **(D)** Place the 3D guide plate and remove the bone along the indicator ring. **(E,F)** Extract the supernumerary teeth and suture the incision.

The surgical site was disinfected with povidone-iodine, and sterile drapes were applied. A sulcular incision was made along the gingival margin of teeth 33–35, with a vertical releasing incision placed mesial to tooth 33. A mucoperiosteal flap was elevated to expose the buccal bone plate. The 3D-printed surgical guide was securely positioned on teeth 34 and 35. Retention relied on occlusal engagement and buccal contour adaptation to teeth 34 and 35; the guide's marginal edges laminated the gingival sulcus without impingement, and stability was verified by inability to dislodge with light surgical manipulation. Bone removal was performed with a high-speed handpiece and 45°angled bur (80 μm, 1.2 mm), following the guide indicators. The supernumerary tooth was completely exposed and removed intact using micro-elevators. The surgical site was irrigated copiously with 0.9% saline solution to remove debris. The flap was repositioned and closed with 4-0 absorbable PGA sutures. Pressure gauze was applied for 40 min to ensure hemostasis. Postoperative Care: The patient was discharged with instructions for oral hygiene maintenance, analgesics (ibuprofen if necessary), and a soft diet. No immediate postoperative complications (e.g., bleeding, infection) were observed (Figure 3). The patient reported resumption of normal oral function within 3 days, with no difficulty in speaking or chewing. The timeline presentation of Case 2 has been presented in Supplementary Table S1.

### Case 3

A 9-year-old male patient presented for consultation after an external radiographic examination revealed two supernumerary teeth in the maxilla. Oral examination: The patient had a mixed dentition with fully erupted permanent teeth 11 and 21, no crowding or malalignment. Soft tissues: No gingival swelling, redness, or ulceration on the palatal side of maxillary anterior teeth; palpation revealed no tenderness or bony prominence. Adjacent teeth: Teeth 11 and 21 showed no mobility, percussion tenderness, or discoloration, indicating no adjacent tissue involvement. CBCT showed a supernumerary tooth with complete root development on the palatal side of tooth 21 and a spherical-shaped supernumerary tooth located apical to the root of tooth 11.

#### Diagnostic reasoning

Initial external radiography detected maxillary anterior radiodense lesions but lacked spatial clarity, prompting CBCT. CBCT confirmed that a fully developed supernumerary tooth (complete crown-root) palatal to 21 and a spherical tuberculate-type one apical to 11—both independent of permanent teeth.

#### Diagnosis

Diagnosis: Two supernumerary teeth on the palatal aspect of teeth 11 and 21.

#### Treatment progress

**Figure 4:**
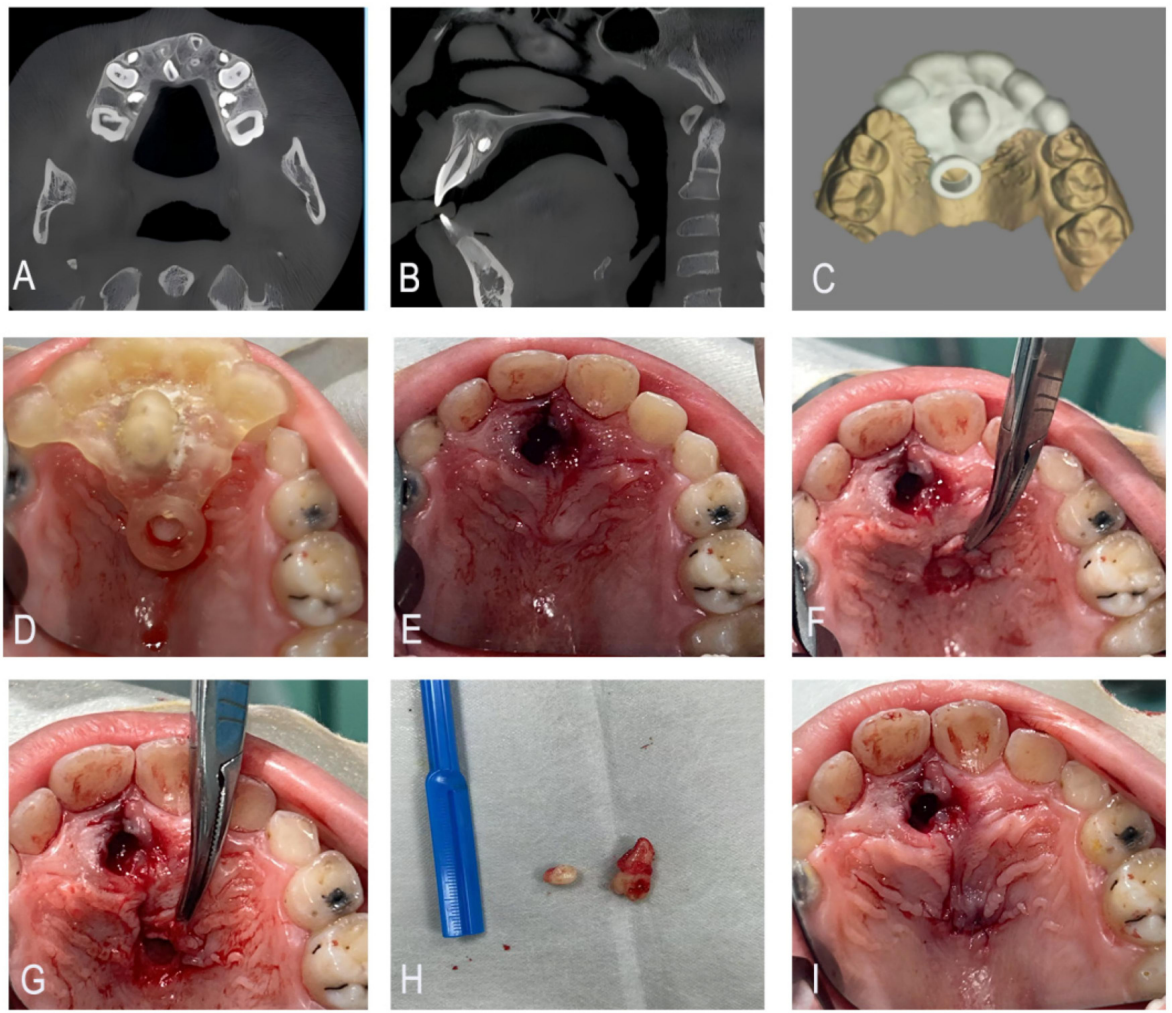
**(A,B)** CBCT showing a fully developed supernumerary tooth on the palatal side of tooth #21 and a spherical supernumerary tooth apical to the root of tooth #11. **(C)** Guide plate designed by 3D printing. **(D–F)** Place the 3D guide plate, flip the flap along the indicator ring and remove the bone covering the surface of the superimposed tooth. **(G–I)** Extract the supernumerary teeth and suture the incision.

The surgical field was disinfected with povidone-iodine, and local infiltration anesthesia was achieved using 4% articaine with 1:100,000 epinephrine. After separating the gingiva with a periosteal elevator, the erupted supernumerary tooth on the palatal side of tooth 21 was extracted using dental forceps. The sterilized 3D-printed surgical guide was then securely positioned on the palatal surface of teeth 11 and 21. Fixation was achieved via palatal contour matching to teeth 11–21—the guide's concave internal surface conformed to the convex palatal anatomy of the incisors, providing sufficient retention to withstand bone removal forces. No loosening was observed during the procedure. An arcuate incision was made along the guide's indicator ring, and a mucoperiosteal flap was elevated to expose the underlying bone plate. Using a high-speed handpiece with a 45-degree angled bur (grain size: 80 μm, diameter: 1.2 mm), bone removal was performed precisely at the predesignated area on the guide. The spherical supernumerary tooth apical to tooth 11 was fully exposed and extracted intact using micro-elevators. The surgical site was irrigated copiously with 0.9% saline solution to remove bone debris. The flap was repositioned and closed with 4-0 absorbable PGA sutures (Figure 4). Pressure gauze was applied for 40 min to achieve hemostasis. The patient was discharged with instructions to maintain oral hygiene, take ibuprofen (10 mg/kg as needed), and follow a soft diet for 3 days. No immediate complications (e.g., active bleeding, soft tissue swelling, or paresthesia) were observed before discharge. No postoperative bleeding, swelling, or paresthesia occurred. The child resumed normal diet and school activities within 1 week. The timeline presentation of Case 3 has been presented in Supplementary Table S1.

The patients were given extended-release ibuprofen for preemptive analgesia 30 min before the procedure, and other surgical preparations were completed. Postoperative pain was relieved with ibuprofen when intolerable. All surgeries were performed under local anesthesia, with 4% articaine with 1:100,000 epinephrine.

All patients reported complete resolution of surgical site swelling within 5–7 days. Suture absorption was confirmed at the 2-week follow-up, with no gingival inflammation or wound dehiscence. At the 1-month follow-up, all patients (or their guardians) expressed satisfaction with the procedure, citing minimal postoperative discomfort and no impact on daily life, which aligned with their preoperative expectations.

For all cases, differential diagnoses included: (1) Odontoma: Ruled out by CBCT showing no fragmented calcified masses or mixed radiodensity, and clear delineation of the supernumerary tooth's crown-root morphology; (2) Dens invaginatus: Excluded as the lesion was an independent structure rather than an invagination of the permanent tooth root; (3) Root fragment: Eliminated by CBCT showing a complete root with apical closure, consistent with a fully developed supernumerary tooth.

## Discussion

The development of supernumerary teeth (ST) is a complex process primarily influenced by genetic factors during embryogenesis (7). These teeth arise from disturbances in the odontogenic process or aberrant proliferation of the dental lamina.

The clinical manifestations of ST vary according to their location, size, and number. Surgical removal is often indicated for impacted ST to prevent complications such as misalignment, root resorption, and adjacent tissue injury (8).

Conventional two-dimensional (2D) radiography has been widely used to detect supernumerary teeth (9). However, 2D imaging cannot adequately display the three-dimensional spatial relationship between ST and adjacent anatomical structures (10), increasing the risk of inadvertent injury to surrounding teeth or neurovascular bundles. In contrast, three-dimensional imaging modalities such as CBCT provide substantial advantages.

CBCT has revolutionized preoperative planning and surgical management of complex dental cases involving impacted or supernumerary teeth (11). It provides high-resolution 3D images, enabling a more accurate assessment of the location, shape, and relationships of supernumerary teeth (9, 12). Against freehand CBCT-guided surgery, the 3D guide eliminated subjective reliance on surgeon interpretation of CBCT images, as shown by minimized bone removal and no adjacent tissue injury in our cases—limitations of freehand techniques highlighted by Gurler et al. (9).

In the present series, CBCT imaging provided valuable information on the spatial relationship of ST to vital structures such as the mental and inferior alveolar nerves, as well as adjacent bone morphology. It also allowed quantitative assessment of critical safety distances (13). Nonetheless, the success of complex procedures such as the removal of impacted ST still depends heavily on the surgeon's expertise and meticulous preoperative planning.

The integration of 3D-printed surgical guides has markedly improved the accuracy and predictability of ST removal procedures (14). By combining CBCT data with 3D scanning of dental models, clinicians can visualize and plan the exact location of the surgical incision, window, and bone removal required for optimal access to the impacted teeth (15). Compared to dynamic surgical navigation system (7), the 3D-guide method in this study avoided reliance on intraoperative real-time imaging and complex navigation hardware, reducing operative setup time and equipment costs. However, navigation systems may offer superior flexibility when encountering unexpected anatomical variations, whereas 3D guides are constrained by their preoperative design. In clinical practice, guide design and fabrication require approximately 8–12 h and cost $80–120 per guide—lower than dynamic navigation systems but higher than freehand surgery. The total cost covers three key components: biocompatible photopolymer resin (≈$20–30 per guide), equipment usage (including maintenance and energy costs, ≈$30–40), and post-processing (isopropyl alcohol cleaning, UV curing, and autoclaving, ≈$30–50). The workflow required 8–12 h from data acquisition to sterilized guide readiness, with breakdowns as follows: CBCT and intraoral scan acquisition (30 min), 3D reconstruction and guide design via Mimics 25.0/3Matic 16.0 (3–4 h, including registration and parameter setting), 3D printing (≈1 h per guide, given a layer height of 50 μm and printing speed of 10 gypsum models/30 min), and post-processing (cleaning: 10 min, UV curing: 20 min, autoclaving: 20 min). This timeline necessitates preoperative scheduling and is longer than freehand surgery. However, advancements in automated design software and high-speed printers can shorten the workflow to 4–6 h, and pre-stored anatomical templates may further reduce design time for common supernumerary tooth locations. Accessibility is enhanced by widespread availability of desktop 3D printers, though it requires basic digital planning training, which may limit adoption in resource-constrained settings.

In this study, we demonstrated that digital 3D-printed guides enable accurate and minimally invasive removal of impacted ST through precise anatomical adaptation. The surgical guide itself was created using a 3D printer, ensuring a highly accurate match with the patient's anatomical structures. The guide was designed to support the bone surface and included a semi-circular opening to expose the supernumerary teeth. The accurate matching of the guide to the patient's dental model enhanced the success of the surgery. Despite their accuracy, the bulk of customized guides may occasionally limit intraoral maneuverability—particularly in pediatric cases. This can be mitigated by reducing guide thickness and refining window design during planning. Additionally, the small sample size (three patients) restricts the generalizability of the findings. While the 3D-printed surgical guide showed favorable outcomes in these cases (minimal trauma, no complications), the results cannot be broadly extrapolated to diverse populations (e.g., different age groups or supernumerary tooth morphologies). This case series aimed to demonstrate technical feasibility rather than confirmed universal efficacy. Future prospective studies with larger cohorts and comparative designs are needed to validate reproducibility across diverse clinical settings.

## Conclusion

The integration of 3D-printed surgical guides in the management of impacted supernumerary teeth provides distinct advantages over conventional methods, particularly regarding accuracy and safety. Combining CBCT imaging with 3D printing enables clinicians to perform highly precise preoperative planning, thereby minimizing surgical risk. The use of 3D-printed guides allows for precise extraction with minimal soft-tissue trauma, leading to faster postoperative recovery.

## Data Availability

The original contributions presented in the study are included in the article/Supplementary Material, further inquiries can be directed to the corresponding author.

## Supplementary material

Supplementary Table S1 Clinical timeline and recovery endpoints for each case.
