# Supplementary material for: Case Report: Minimally invasive removal of impacted supernumerary teeth using digital 3D-printed surgical guides: a case series
Source: Front Surg. 2025 Nov 17;12:1653013. doi: 10.3389/fsurg.2025.1653013 (PMC12665692; doi:10.3389/fsurg.2025.1653013)
Supplement: Supplementary Table S1 — Clinical timeline and recovery endpoints for each case. [file Table1.docx]

**Clinical Timeline and Recovery Endpoints for Each Case**

| **Timeline Event / Endpoint** | **Case 1 (20y, female, Mandibular premolars)** | **Case 2 (13y, female, Mandibular 34-35)** | **Case 3 (9y, male, Maxillary incisors)** |
| --- | --- | --- | --- |
| **Initial Diagnosis (CBCT)** | June 7th, 2024 | July 22th, 2024 | July 6th, 2024 |
| **Surgical Intervention** | July 11th, 2024 | July 30th, 2024 | July 8th, 2024 |
| **Resolution of Swelling** | 4 days post-op | 3 days post-op | 3 days post-op |
| **Wound Healing Endpoint (Clinical)** | **2 weeks post-op:** - Complete mucosal re-epithelialization. - No inflammation, dehiscence, or infection. - Suture absorption confirmed. | **2 weeks post-op:** - Complete mucosal re-epithelialization. - No inflammation, dehiscence, or infection. - Suture absorption confirmed. | **2 weeks post-op:** - Complete mucosal re-epithelialization. - No inflammation, dehiscence, or infection. - Suture absorption confirmed. |
| **Functional Recovery Endpoint** | **3 days post-op:** - Normal speech and masticatory function restored with soft diet. - No pain/discomfort during function. | **3 days post-op:** - Normal speech and masticatory function restored with soft diet. - No pain/discomfort during function. | **1week post-op:** - Full return to normal diet and activities. - No avoidance of chewing on surgical site. |
| **Final Follow-up / Satisfaction Assessment** | 1 month post-op | 1 month post-op | 1 months post-op |
